# Presynaptic Cell Dependent Modulation of Inhibition in Cortical Regions

**DOI:** 10.2174/157015909788848875

**Published:** 2009-06

**Authors:** Afia B Ali

**Affiliations:** University of London, School of Pharmacy, Department of Pharmacology 29/39 Brunswick Square, London WC1N 1AX, UK

**Keywords:** Presynaptic, inhibition, cortical, GABA_A_, cannabinoid receptor type-1 (CB1), kainate receptors, mGluRs, depolarisation induced suppression of inhibition (DSI).

## Abstract

Several lines of evidence suggest that the modulation of presynaptic GABA release is mediated by a variety of receptors including; presynaptic AMPA, cannabinoid, GABAB, kainate, metabotropic glutamate, NMDA, and opioid receptors. The evidence supporting presynaptic modulation of inhibition is predominantly obtained from studying stimulus elicited, spontaneous or miniature synaptic events, where the information regarding the identity of the presynaptic cell is lost. This article summarises these findings then focuses on another approach to study the presynaptic modulation of GABA release by comparing the modulation of GABA release at unitary synapses identified morphologically, immunocytochemically and electrophysiologically. To date, evidence for cell-type specific regulation of presynaptic inhibition at identified synapses involving most of the above presynaptic receptors does not exist. Therefore, the key presynaptic modulators that will be focused on here are kainate and cannabinoid receptors and their intracellular signalling cascades that orchestrate GABA release. There will be some discussion on presynaptic modulation *via* opioid receptors at identified synapses. This review provides evidence to suggest a cell-type specific modulation of presynaptic inhibition in cortical regions.

## INTRODUCTION

Inhibition is essential in shaping response properties in single cells and assisting co-operativtity in large populations of cells. It is the network of GABAergic interneurones that balances excitability by controlling dendritic electrogenesis and spike generation of pyramidal cells as well as setting and maintaining oscillatory rhythms. In CA1 alone there are currently 22 identified subclasses of interneurones [54]. These interneurones are classified according to their neurochemistry, electrophysiological properties and their gross morphology [32, 51, 62, 81, 84]. In the neocortex and hippocampus, inhibitory interneurones may terminate on pyramidal cell dendrites, these include bistratified cells [5, 12] Schaffer collateral associated interneurones [3, 17, 72, 93] and oriens lacunosum molecular interneurones in the hippocampus, [7-9, 81] and Martinotti, double bouquet, bipolar and bitufted cells in the neocortex [51, 85, 94]. These dendrite-preferring interneurones serve to fine-tune pyramidal cell activity by allowing a wide time window for coincidence detection [65].

Other interneurones target proximal regions of pyramidal cells, for example basket cells [4, 12, 81] and axo-axonic cells [56, 85]. These cells are thought to have a functional role in negating pyramidal cell activity by responding faster and more reliably, thus restricting the time window for spike generation in the postsynaptic target cells [15, 34, 48, 65, 73]. However, the properties of proximally targeting basket cells are becoming well documented [see 33, 34 for reviews] and their role in “negating pyramidal cell” activity is far more complex because basket cells are active at different times during network behaviour [53, 54]. There are two different types of basket cells; parvalbumin and cholecystokinin (CCK) –positive cells. The most widely distributed are parvalbumin positive, cells which usually display fast, non-accommodating action potentials, with fast membrane time constants and elicit fast inhibitory postsynaptic potentials mediated predominantly by alpha 1 subunit containing GABA_A _receptors [4, 5, 8, 88]. These fast properties allow the fast spiking basket cells to faithfully respond to repetitive excitation [5, 38]. Conversely, CCK-positive basket cells usually display slower, accommodating action potentials and membrane time constants and elicit slower IPSPs that are mediated by alpha 2/3 subunit containing GABA_A_ receptors [5, 8, 38, 88]. Thus, although structurally basket cells target similar postsynaptic domains, there is dichotomy in the physiological functions.

Selective insertion of presynaptic receptors such as kainate, cannabinoid (CB), AMPA, NMDA, GABA_B_, opioid, and metabotropic glutamate receptors (mGluRs) may add further diversity to interneuronal function. These signalling pathways exert a modulatory role on transmitter release at inhibitory synapses, though how all these receptors/modulators variously regulate inhibition in a cell type-specific manner still requires detailed investigation. The evidence surrounding presynaptic modulation of inhibition at identified synapses available to date predominantly focuses on presynaptic basket cells, probably because the CCK-positive basket cells express a range of presynaptic receptors and modulators, such as; CB1 and 5-HT3 receptors, vesicular glutamate transporter type 3 and a high level of GABA_B_ receptors [see 31, 32, 83 for reviews]. Parvalbumin basket cells express fewer presynaptic modulators/receptors, therefore the different physiological properties of these two subclasses of basket cells could be a reflection of their diverse intrinsic properties, as well as the modulatory pathways involved. Here, I will be reviewing these two subclasses of basket cells, and dendrite targeting cells that express CCK and focus on presynaptic modulation *via* kainate and cannabinoid receptors with some discussion on opioid receptors.

### Modulation of GABA *Via* Presynaptic Kainate Receptors

Cortical interneurones mostly express the GluR5 or GluR6 subunits of kainate receptors [13, 71].

**Fig. (1):**
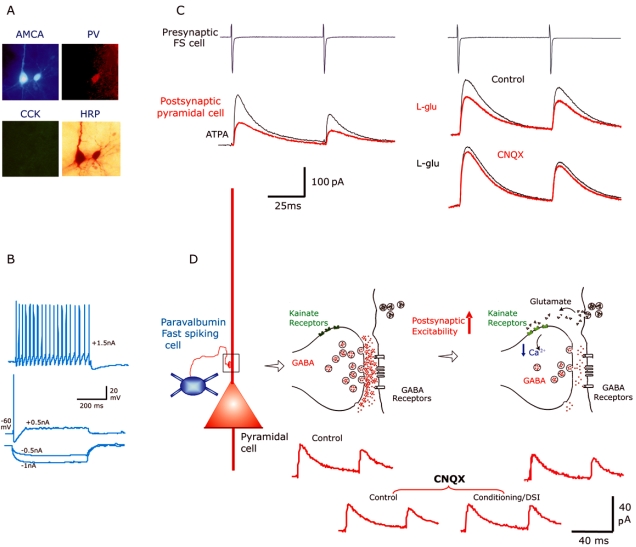
Presynaptic kainate receptors regulate unitary IPSPs in rat neocortex. (**A**) Pair of synaptically connected cells labelled with biocytin (marked with AMCA). The postsynaptic interneurone was immuno-positive for Parvalbumin (PV) and negative for cholecystokinin- (CCK). This type of multipolar basket cells typically display fast firing patterns (**B**). (**C**) Voltage clamp recordings to demonstrate the suppression of unitary IPSCs elicited by fast spiking interneurones in pyramidal cells in control, during bath application of ATPA (1 µ**M**, GluR5 specific agonist) and during L-glutamate (10µ**M**) . Subsequent addition of CNQX (30 µ**M**, broad spectrum, AMPA and kainate receptor antagonist) almost completely abolished the suppression of these IPSCs. (**D**) Schematic diagram illustrating a synapse between a fast spiking interneurone and a pyramidal cell in the neocortex. The endogenous release of L-glutamate as a retrograde messenger from the postsynaptic pyramidal cell as a result of depolarisation (increase in postsynaptic excitation i.e. conditioning protocol) is thought to have suppressed the IPSCs as result of activating presynaptic kainate receptors. This suppression was prevented by CNQX [See ref. 6 for further details].

The first evidence to suggest that kainate receptors decrease inhibition came from studies performed in the hippocampus [25, 82, 96]. Following these studies, it has been demonstrated that kainate acts presynaptically to modulate the release of neurotransmitter release [see 46, 55  for reviews]. Evidence of presynaptic kainate receptors regulating inhibition came from stimulus elicited experiments where inhibitory postsynaptic currents (IPSCs) were shown to be depressed with either exogenous application of kainate [13, 19, 29, 30, 74, 75, 76] or by a direct activation of glutamatergic pathways [66]. However, some interneurones including fast spiking basket cells (that have been so far identified) also express functional somato-dendritic kainate receptors, which upon activation will enhance the spontaneous firing rates of these cells, therefore increasing the frequency of spontaneous IPSCs [13, 29, 30]. This has lead to the assumption that the depression of stimulus elicited IPSCs during application of kainate receptor agonists could be due to the secondary effects of excess GABA acting on both pre- and postsynaptic cells rather than the activation of presynaptic kainate receptors [29, 30]. One way to demonstrate the involvement of kainate receptors on presynaptic terminals was to look at action potential independent spontaneous IPSCs (or miniature IPSCs). However, although some studies have reported a decrease in the frequency of miniature IPSC [21] others have disagreed [13, 29, 30]. Thus there was debate as to whether kainate receptors are located presynaptically to modulate inhibition. More direct evidence was provided by studying unitary IPSCs elicited by fast spiking interneurones (which are typically immunoreactive for Parvalbumin) in layer V pyramidal cells of the neocortex. IPSCs were depressed by ATPA, a GluR5 kainate receptor subunit specific agonist and by the endogenous agonist L-glutamate (in the presence of AMPA, NMDA, mGluR and GABA_B_ receptor antagonists) suggesting the involvement of the GluR5 subunit [6]. This is illustrated in (Fig. **1**). These effects were accompanied by an increase in the failure rate of synaptic transmission, in the coefficient of variation and in the paired pulse ratio, indicating a presynaptic origin of the IPSC depression.

Some studies have suggested that presynaptic kainate receptors increase, rather than inhibit GABA release at connections between inhibitory neurones in CA1 [19, 47, 67], in hypothalamic neurones [57] and in cultured dorsal horn neurons [52]. Interestingly this bidirectional role for kainate receptor modulation of inhibition has been reported in the amygdale [11]. The exact mechanisms that enhance GABA release at these synapses remains unclear.

These data suggest that the modulation of GABA mediated synaptic events is heterogeneous and dependent on the postsynaptic target neurone. There is sufficient evidence to suggest that within cortical regions kainate receptors reduce the inhibitory efficacy of synapses presynaptically.

### Intracellular Signalling Cascades Involved in Activating Presynaptic Kainate Receptor

Intracellular signalling cascades that trigger presynaptic kainate receptors probably co-operate with postsynaptic mechanisms such as depolarisation induced suppression of inhibition (DSI). This mechanism is triggered by postsynaptic membrane depolarisation and requires the opening of voltage dependent calcium channels in the postsynaptic cell, resulting in a release of retrograde signal to act on inhibitory interneurones presynaptically to reduce the release of GABA [1, 2, 64]. Previous studies have suggested that glutamate is released from postsynaptic dendrites as a result of DSI acting as a retrograde messenger [6, 100].This glutamate then activates presynaptic kainate receptors that probably inactivate presynaptic calcium channels, hence a reduced influx of calcium decreasing GABA release [49, 60, 78]. These regulatory mechanisms also may involve G-proteins since a decrease in GABA release induced by kainate receptors is affected by PTx-sensitive G-protein and phosphykinase C activation [75, 76]. 

### Modulation of GABA Release *Via* Cannabinoid Receptors

Cannabinoid receptors constitute a major family of G protein- coupled receptors. There are two major types, CB1 and CB2, of which CB1 is predominantly found in the CNS [for reviews see, 39, 41, 45, 77, 97].

In the CNS, CB1 receptor mRNA is predominantly localised in neocortical and hippocampal presynaptic terminals in subsets of GABAergic interneurones [43, 50, 63, 89]. In particular, axon terminals of CB1 receptors were also co-localised with cholecystokinin (CCK), but never parvalbumin shown by double immuno-labelling experiments [10, 39, 50, 68]. This is also supported by physiological data. The first intracellular recordings that provided evidence for the modulation of GABAergic synaptic transmission *via* CB1 receptors were from hippocampal pyramid cells, *in-vitro*. IPSCs were reduced by bath application of an exogenous cannabinoid without affecting the action potential independent spontaneous events, supporting the presynaptic site of cannabinoid action [40, 42, 43]. This modulation of GABA release is absent in CB1 receptor knockout mice [40, 69, 98]. In the neocortex for example, inhibitory potentials elicited by CB1 receptor-expressing, regular spiking interneurones (but not fast spiking interneurones) to pyramidal cells connections are suppressed by endocannabinoids [35].

**Fig. (2):**
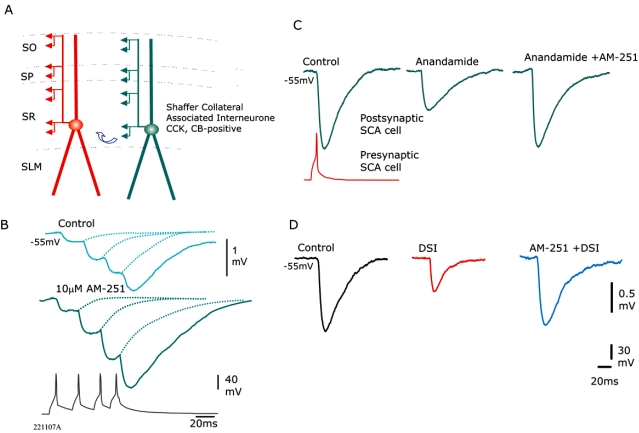
Inhibition at CCK-positive terminals is regulated by CB1 receptors. (**A**) Schematic of 2 connected CCK-positive Shaffer collateral associated interneurones (SCA) in CA1 stratum radiatum. These connections typically displayed synaptic facilitation (**B**) and were sensitive to CB1 receptor pharmacology (**C**) and depolarisation induced suppression of inhibition (DSI) (**D**). CB1 receptor antagonist/inverse agonist AM-251 (10 µ**M**) prevented the actions of Anandamide (14 µM, CB receptor agonist) and DSI [see ref. 3 for further details]. (**B**) Bath application of AM-251 at another connection between 2 SCA interneurones resulted in an enhancement of the train of IPSPs elicited. This enhancement to the train of IPSPs suggests that there is a persistent silencing of these synapses *via* CB1 receptors that was relieved by AM-251.

Co-localisation of CCK and CB1 receptors are not restricted to proximally targeting basket cells and are also expressed by dendrite targeting interneurones [see 3, 17, 32, 54, 72, 93]. Hence, there is some overlap of CB1 receptor function with perisomatic and dendritic inhibition which is discussed below. In CA3, CCK-positive mossy-fiber-associated interneurones contact apical dendrites of pyramidal cells. Using paired whole-cell recordings, an increased firing rate of this presynaptic interneurone relieved silencing of this synapse by persistent CB1 receptor activation [58]. This observation was extended to CA1, where GABA release at CCK-positive basket cells targeting pyramidal cells was decreased *via* presynaptic CB1 receptors at low frequencies of presynaptic firing, however the presynaptic basket cell recovered from this inhibition of GABA release when it was activated at higher firing frequencies [27]. These synapses showed a tonic silencing which has been suggested to be a result of tonic endocannabinoid mobilization from postsynaptic pyramidal cells and this release of endocannabinoids is thought to be regulated by mGluRs and muscarinic receptors [26]. Recently, these observations have extended to unitary connections among interneurones [3]. We have observed that there is a target-cell dependent short-term synaptic plasticity of IPSPs elicited by presynaptic CCK-positive cells onto a variety of postsynaptic interneurones that could also indirectly play an important role in spike timing of pyramidal cells. These synapses all modulate their GABA release *via* presynaptic CB1 receptors that may alter their short-term dynamics [3, Ali and Todorova, unpublished observations]. (Fig. **2**) illustrates IPSPs between SCA interneurones which are reduced by a CB receptor agonist, Anandamide. The decrease of GABA release was prevented by the CB1 receptor antagonist AM-251. Fig. (**2B**) also demonstrates there is a persistent inhibition due to activation of CB1 receptors at these connections that was relieved after the bath application of AM-251, resulting in an enhancement of the unitary IPSPs (Ali and Todorova, unpublished observation). This persistent silencing *via* CB1 receptors probably plays a role in differentiating between certain ensembles of pyramidal cells, allowing ongoing activity at some ensembles, while silencing others. In contrast, the persistent silencing *via* CB1 receptors observed in hippocampal regions is not always present at neocortical inhibitory connections involving presynaptic regular spiking interneurones (De-May and Ali, unpublished observations), suggesting that persistent silencing *via* CB1 receptors may be predominantly a hippocampal function.

In summary, there is a consistent observation that GABA release is modulated *via* CB1 receptors at synapses that co-localise CCK and CB1 receptors. This probably contributes to short and long term synaptic plasticity throughout the brain. Interestingly, CCK, itself is a modulator at synapses by acting as molecular switch that determines the source of perisomatic inhibition [26]. The study by Földy and colleagues demonstrates that CCK selectively excites and enhances the output of parvalbumin-expressing basket cells, while concurrently suppressing GABA release from CCK-positive basket cells. It has been suggested that this reduction is triggering endocannabinoid mediated, retrograde signalling, since CCK-B receptors are linked to G-protein coupled receptors that can act through phospholipase C [95] leading to endocannabinoid production and release.

### Intracellular Signalling Cascades that Trigger Down-Regulation of Inhibition Via CB1 Receptors

Much of the attention focused on endogenous cannabinoids as a retrograde signal is linked with DSI (a decrease in GABA release presynaptically as a result of postsynaptic membrane depolarisation) due to the following observation; firstly exogenous cannabinoids modulate GABA release [3, 21, 24, 28, 35, 37, 69, 98]. Secondly both DSI and endocannbinoid synthesis require Ca^2+^ influx into the postsynaptic cell. Thirdly, DSI expression is thought to be presynaptic since it does not affect the quantal size of miniature GABA mediated events [2, 97] consistent with the presynaptic location of cannabinoid receptors. Most studies reporting the modulation of synapses *via* CB1 receptors usually employ the DSI protocols, however in the neocortex, connections between CB1-positive cells lack DSI [35] suggesting that perhaps another CB receptor is involved here.

When endocannabinoids are released they activate CB1 receptors to modulate neuronal signalling mainly *via* the inhibition of adenylate cyclase and N and P/Q type calcium channels [23, 60, 70, 90, 98] or by activation of inwardly rectifying potassium channels [20, 79, 92]. The inhibition of presynaptic calcium channels could result in the suppression of the release of neurotransmitters such as glutamate, acetylcholine, noradrenaline and GABA [36, 39, 42, 44, 50].

The presynaptic activation of CB1 receptors is most likely linked to an inhibition of N- or P/Q-type voltage gated calcium channels involved in vesicular release [42, 90]. Recently it has been suggested that mGluR1 effects on DSI may be a result of the activation of endocannabinoids with glutamate acting as a trigger rather than as a retrograde signal in the cerebellum [61, 91].

## MODULATION OF GABA RELEASE *VIA* OPIOID RECEPTORS

Opioids are powerful modulators of inhibition in the hippocampus [14, 16, 18, 80, 99] where these receptors strongly increase the spiking probability of pyramidal cells [59]. It has been demonstrated that opioids selectively suppress inhibition on parvalbumin-positive, fast spiking basket cells, but not regular spiking, CCK-positive basket cells [37]. The study by Glickfeld and colleagues suggests that opioids modulate the membrane potential of fast spiking basket cells is consistent with the evidence that opioid-mediated outward currents result from both the opening and closing of hyperpolarizing and depolarizing conductances, respectively [87]. These results are consistent with the preferential colocalization of opioid receptors with parvalbumin but not with CCK in synaptic terminals [22, 86].

## CONCLUSION

This review focuses on the regulatory mechanisms of identified subclasses of basket and other CCK-positive cells and their presynaptic inhibition involving kainate and cannabinoid receptors. The cascade of events leading to presynaptic mechanisms regulating GABA release is probably a dynamic process with postsynaptic mechanisms. These presynaptic modulatory pathways are also strongly correlated with the class of presynaptic interneurones recruited and perhaps there is a selective insertion of presynaptic receptors. Subclasses of basket cells illustrate this well, for example, CCK-positive basket cells have a great array of presynaptic receptors and modulators in comparison to the parvalbumin-positive basket cells, specializing CCK-basket cells to be highly modifiable allowing fine-tuning of perisomatic inhibition. Perhaps parvalbumin basket cells that have a more rigid and precise nature of inhibition in synchronizing the network [15, 53] do not use many modulatory pathways that fine-tune inhibition because it is not required.

Thus selective modulation by activity-dependent release of neurotransmitter *via* specific presynaptic receptors may change the strength and properties of inhibition in cortical regions. These modulatory pathways may also act in a complementary manner that regulates these two distinct sources of inhibition in a co-ordinated, but opposing manner by amplifying one source and dampening inhibition on another.

Future experiments need to focus on revealing how the many presynaptic receptors and modulators regulate synaptic strength at other identified subclasses of interneurones. With the existence of diverse inhibitory circuitry in the cortical regions, it is of interest to dissect how these interneurones and the pre and postsynaptic mechanisms involved in mediating responses determine the overall effect of synaptic inhibition.

## References

[1] Alger BE (2002). Retrograde signalling in the regulation of synaptic transmission: focus on endocannabinoids. Prog.Neurobiol.

[2] Alger BE, Pitler TA (1995). Retrograde signaling at GABA A-receptor synapses in the mammalian CNS. Trends Neurosci.

[3] Ali AB (2007). Presynaptic Inhibition of GABAA receptor-mediated unitary IPSPs by cannabinoid receptors at synapses between CCK-positive interneurons in rat hippocampus. J.Neurophysiol.

[4] Ali AB, Bannister AP, Thomson AM (1999). IPSPs elicited in CA1 pyramidal cells by putative basket cells in slices of adult rat hippocampus. Eur.J. Neurosci.

[5] Ali AB, Deuchars J, Pawelzik H, Thomson AM (1998). CA1 pyramid to basket and bistratified cells EPSPs: dual intracellular recordings in rat hippocampal slices. J.Physiol.

[6] Ali AB, Rossier J, Staiger JF, Audinat E (2001). Kainate receptors regulate unitary IPSCs elicited in pyramidal cells by fast-spiking interneurons in the neocortex. J. Neurosci.

[7] Ali AB, Thomson AM (1998). Facilitating pyramid to horizontal oriens-alveus interneurone inputs: dual intracellular recordings in slices of rat hippocampus. J. Physiol.

[8] Ali AB, Thomson AM (2008). Synaptic alpha 5 subunit-containing GABAA receptors mediate IPSPs elicited by dendrite-preferring cells in rat neocortex. Cereb. Cortex.

[9] Blasco-Ibáñez JM, Freund TF (1995). Synaptic input of horizontal interneurons in stratum oriens of the hippocampal CA1 subfield: structural basis of feed-back activation. Eur. J. Neurosci.

[10] Bodor AL, Katona I, Nyíri G, Mackie K, Ledent C, Hájos N, Freund TF (2005). Endocannabinoid signaling in rat somatosensory cortex: laminar differences and involvement of specific interneuron types. J. Neurosci.

[11] Braga MF, Aroniadou-Anderjaska V, Xie J, Li H (2003). Bidirectional modulation of GABA release by presynaptic glutamate receptor 5 kainate receptors in the basolateral amygdala. J. Neurosci.

[12] Buhl EH, Halasy K, Somogyi P (1994). Diverse sources of hippocampal unitary inhibitory postsynaptic potentials and the number of synaptic release sites. Nature.

[13] Bureau I, Bischoff S, Heinemann SF, Mulle C (1999). Kainate receptor-mediated responses in the CA1 field of wild-type and GluR6-deficient Mice. J. Neurosci.

[14] Capogna M, Gahwiler BH, Thompson SM (1993). Mechanism of mu-opioid receptor-mediated presynaptic inhibition in the rat hippocampus *in vitro*. J. Physiol.

[15] Cobb SR, Buhl EH, Halasy K, Paulsen O, Somogyi P (1995). Synchronization of neuronal activity in hippocampus by individual GABAergic interneurons. Nature.

[16] Cohen GA, Doze VA, Madison DV (1992). Opioid inhibition of GABA release from presynaptic terminals of rat hippocampal interneurons. Neuron.

[17] Cope DW, Maccaferri G, Marton LF, Roberts JD, Cobden PM, Somogyi P (2002). cholecystokinin-immunopositive basket and Schaffer collateral-associated interneurones target different domains of pyramidal cells in the CA1 area of the rat hippocampus. Neuroscience.

[18] Corrigall WA, Linseman MA (1980). A specific effect of morphine on evoked activity in the rat hippocampal slice. Brain Res.

[19] Cossart R, Esclapez M, Hirsch JC, Bernard C, Ben-Ari Y (1998). GluR5 kainate receptor activation in interneurones increase tonic inhibition of pyramidal cells. Nat. Neurosci.

[20] Daniel H, Crepel F (2001). Control of Ca^(2+)^ influx by cannabinoid and metabotropic glutamate receptors in rat cerebellar cortex requires K(+) channels. J. Physiol.

[21] Diana MA, Marty A (2004). Endocannabinoid-mediated short-term synaptic plasticity: depolarization-induced suppression of inhibition (DSI) and depolarization-induced suppression of excitation (DSE). Br. J. Pharmacol.

[22] Drake CT, Milner TA (2002). Mu opioid receptors are in discrete hippocampal interneuron subpopulations. Hippocampus.

[23] Felder CC, Joyce KE, Briley EM, Mansouri J, Mackie K, Blond O, Lai Y, Ma AL, Mitchell RL (1995). Comparison of the pharmacology and signal transduction of the human cannabinoid CB1 and CB2 receptors. Mol. Pharmacol.

[24] Ferraro L, Tomasini MC, Cassano T, Bebe BW, Siniscalchi A, O'Connor WT, Magee P, Tanganelli S, Cuomo V, Antonelli T (2001). Cannabinoid receptor agonist WIN 55,212-2 inhibits rat cortical dialysate gamma-aminobutyric acid levels. J. Neurosci. Res.

[25] Fisher RS, Alger BE (1984). Electrophysiological mechanisms of kanic acid-induced epeliptiform activity in the rat hippocampal slices. J. Neurosci.

[26] Földy C, Neu A, Jones MV, Soltesz I (2007). Postsynaptic origin of CB1-dependent tonic inhibition of GABA release at cholecystokinin-positive basket cell to pyramidal cell synapses in the CA1 region of the rat hippocampus. J. Physiol.

[27] Földy C, Neu A, Jones MV, Soltesz I (2006). Presynaptic, activity-dependent modulation of cannabinoid type 1 receptor-mediated inhibition of GABA release. J. Neurosci.

[28] Fortin DA, Levine ES (2007). Differential effects of endocannabinoids on glutamatergic and GABAergic inputs to layer 5 pyramidal neurons. Cereb. Cortex.

[29] Frerking M, Malenka RC, Nicoll RA (1998). Synaptic activation of kainate receptors on hippocampal interneurones. Nat. Neurosci.

[30] Frerking M, Petersen CC, Nicoll RA (1999). Mechanisms underlying kainate receptor-mediated disinhibition in the hippocampus. Proc. Natl. Acad. Sci. USA.

[31] Freund TF (2003). Interneuron diversity series: rhythm and mood in perisomatic inhibition. Trends Neurosci.

[32] Freund TF, Buzsaki G (1996). Interneurons of the hippocampus. Hippocampus.

[33] Freund TF, Katona I (2007). Perisomatic inhibition. Neuron.

[34] Fricker D, Miles R (2001). Interneurons, spike timing, and perception. Neuron.

[35] Galarreta M, Erdélyi F, Szabó G, Hestrin S (2008). Cannabinoid sensitivity and synaptic properties of 2 GABAergic networks in the neocortex. Cereb. Cortex.

[36] Gifford AN, Ashby CR Jr (1996). Electrically evoked acetylcholine release from hippocampal slices is inhibited by the cannabinoid receptor agonist, WIN 55212-2, and is potentiated by the cannabinoid antagonist, SR 141716A. J. Pharmacol. Exp. Ther.

[37] Glickfeld LL, Atallah BV, Scanziani M (2008). Complementary modulation of somatic inhibition by opioids and cannabinoids. J. Neurosci.

[38] Glickfeld LL, Scanziani M (2006). Distinct timing in the activity of cannabinoid-sensitive and cannabinoid-insensitive basket cells. Nat. Neurosci.

[39] Hájos N, Freund TF (2002). Distinct cannabinoid sensitive receptors regulate hippocampal excitation and inhibition. Chem. Phys. Lipids.

[40] Hajos N, Katona I, Naiem SS, MacKie K, Ledent C, Mody I, Freund TF (2000). Cannabinoids inhibit hippocampal GABAergic transmission and network oscillations. Eur. J. Neurosci.

[41] Hampson RE, Deadwyler SA (1999). Cannabinoids, hippocampal function and memory. J. Life Sci.

[42] Hoffman AF, Lupica CR (2000). Mechanisms of cannabinoid inhibition of GABA(A) synaptic transmission in the hippocampus. J. Neurosci.

[43] Irving AJ, Coutts AA, Harvey J, Rae MG, Mackie K, Bewick GS, Pertwee RG (2000). Functional expression of cell surface cannabinoid CB(1) receptors on presynaptic inhibitory terminals in cultured rat hippocampal neurons. Neuroscience.

[44] Ishac EJ, Jiang L, Lake KD, Varga K, Abood ME, Kunos G (1996). Inhibition of exocytotic noradrenaline release by presynaptic cannabinoid CB1 receptors on peripheral sympathetic nerves. Br. J. Pharmacol.

[45] Iversen L (2003). Cannabis and the brain. Brain.

[46] Jaskolski F, Coussen F, Mulle C (2005). Subcellular localization and trafficking of kainate receptors. Trends Pharmacol. Sci.

[47] Jiang L, Xu J, Nedergaard M, Kang J (2001). A kainate receptor increases the efficacy of GABAergic synapses. Neuron.

[48] Jonas P, Bischofberger J, Fricker D, Miles R (2004). Interneuron Diversity series: Fast in, fast out--temporal and spatial signal processing in hippocampal interneurons. Trends Neurosci.

[49] Kamiya H, Ozawa S (1998). Kainate receptor-mediated inhibition of presynaptic Ca 21 influx and EPSP in area CA1 of the rat hippocampus. J.Physiol.

[50] Katona I, Sperlágh B, Sík A, Käfalvi A, Vizi ES, Mackie K, Freund TF (1999). Presynaptically located CB1 cannabinoid receptors regulate GABA release from axon terminals of specific hippocampal interneurons. J. Neurosci.

[51] Kawaguchi Y, Kubota Y (1997). GABAergic cell subtypes and their synaptic connections in rat frontal cortex. Cereb. Cortex.

[52] Kerchner GA, Wang GD, Qiu CS, Huettner JE, Zhuo M (2001). Direct presynaptic regulation of GABA/glycine release by kainate receptors in the dorsal horn: an ionotropic mechanism. Neuron.

[53] Klausberger T, Marton LF, O'Neill J, Huck JH, Dalezios Y, Fuentealba P, Suen WY, Papp E, Kaneko T, Watanabe M, Csicsvari J, Somogyi P (2005). Complementary roles of cholecystokinin- and parvalbumin-expressing GABAergic neurons in hippocampal network oscillations. J. Neurosci.

[54] Klausberger T, Somogyi P (2008). Neuronal diversity and temporal dynamics: the unity of hippocampal circuit operations. Science.

[55] Lerma J (2003). Roles and rules of kainate receptors in synaptic transmission. Nat. Rev. Neurosci.

[56] Li XG, Somogyi P, Tepper JM, Buzsáki G (1992). Axonal and dendritic arborization of an intracellularly labeled chandelier cell in the CA1 region of rat hippocampus. Exp. Brain Res.

[57] Liu QS, Patrylo PR, Gao XB, van den Pol AN (1999). Kainate acts at presynaptic receptors to increase GABA release from hypothalamic neurons. J. Neurophysiol.

[58] Losonczy A, Biró AA, Nusser Z (2004). Persistently active cannabinoid receptors mute a subpopulation of hippocampal interneurons. Proc. Natl. Acad. Sci. USA.

[59] Lupica CR, Dunwiddie TV (1991). Differential effects of mu- and delta-receptor selective opioid agonists on feedforward and feedback GABAergic inhibition in hippocampal brain slices. Synapse.

[60] Mackie K, Hille B (1992). Cannabinoids inhibit N-type calcium channels in neuroblastoma-glioma cells. Proc. Natl. Acad. Sci. USA.

[61] Maejima T, Ohno-Shosaku T, Kano M (2001). Endogenous cannabinoid as a retrograde messenger from depolarized postsynaptic neurons to presynaptic terminals. Neurosci. Res.

[62] Markram H, Toledo-Rodriguez M, Wang Y, Gupta A, Silberberg G, Wu C (2004). Interneurons of the neocortical inhibitory system. Nat. Rev. Neurosci.

[63] Marsicano G, Lutz B (1999). Expression of the cannabinoid receptor CB1 in distinct neuronal subpopulations in the adult mouse forebrain. Eur. J. Neurosci.

[64] Marty A, Llano I (1995). Modulation of inhibitory synapses in the mammalian brain. Curr. Opin. Neurobiol.

[65] Miles R, Tóth K, Gulyás AI, Hájos N, Freund TF (1996). Differences between somatic and dendritic inhibition in the hippocampus. Neuron.

[66] Min MY, Melyan Z, Kullmann DM (1999). Synaptically released glutamate reduces gamma-aminobutyric acid (GABA)ergic inhibition in the hippocampus *via* kainate receptors. Proc. Natl. Acad. Sci. USA.

[67] Mulle C, Sailer A, Swanson GT, Brana C, O'Gorman S, Bettler B, Heinemann SF (2000). Subunit composition of kainate receptors in hippocampal interneurons. Neuron.

[68] Nyíri G, Szabadits E, Cserép C, Mackie K, Shigemoto R, Freund TF (2005). GABAB and CB1 cannabinoid receptor expression identifies two types of septal cholinergic neurons. Eur. J. Neurosci.

[69] Ohno-Shosaku T, Maejima T, Kano M (2001). Endogenous cannabinoids mediate retrograde signals from depolarized postsynaptic neurons to presynaptic terminals. Neuron.

[70] Pan X, Ikeda SR, Lewis DL (1996). Rat brain cannabinoid receptor modulates N-type Ca^2+^ channels in a neuronal expression system. Mol. Pharmacol.

[71] Paternain AV, Herrera MT, Nieto MA, Lerma J (2000). GluR5 and GluR6 kainate receptor subunits coexist in hippocampal neurons and coassemble to form functional receptors. J. Neurosci.

[72] Pawelzik H, Hughes DI, Thomson AM (2002). Physiological and morphological diversity of immunocytochemically defined parvalbumin- and cholecystokinin-positive interneurones in CA1 of the adult rat hippocampus. J. Comp. Neurol.

[73] Pouille F, Scanziani M (2001). Enforcement of temporal fidelity in pyramidal cells by somatic feed-forward inhibition. Science.

[74] Rodriguez-Moreno A, Herreras O, Lerma J (1997). Kainate receptors presynaptically downregulate GABAergic inhibition in the rat hippocampus. Neuron.

[75] Rodriguez-Moreno A, Lerma J (1998). Kainate receptor modulation of GABA release involves a metabotropic function. Neuron.

[76] Rodriguez-Moreno A, Lopez-Garcia JC, Lerma J (2000). Two populations of kainate receptors with separate signaling mechanisms in hippocampal interneurons. Proc. Natl. Acad. Sci. USA.

[77] Safo PK, Cravatt BF, Regehr WG (2006). Retrograde endocannabinoid signaling in the cerebellar cortex. Cerebellum.

[78] Schmitz D, Frerking M, Nicoll RA (2000). Synaptic activation of presynaptic kainate receptors on hippocampal mossy fiber synapses. Neuron.

[79] Schweitzer P (2000). Cannabinoids decrease the K(+) M-current in hippocampal CA1 neurons. J. Neurosci.

[80] Siggins GR, Zieglgansberger W (1981). Morphine and opioid peptides reduce inhibitory synaptic potentials in hippocampal pyramidal cells *in vitro* without alteration of membrane potential. Proc. Natl. Acad. Sci. USA.

[81] Sik A, Penttonen M, Ylinen A, Buzsáki G (1995). Hippocampal CA1 interneurons: an *in vivo* intracellular labeling study. J. Neurosci.

[82] Sloviter RS, Damiano BP (1981). On the relationship between kainic acid-induced epileptiform activity and hippocampal neuronal damage. Neuropharmacology.

[83] Somogyi P, Tamás G, Lujan R, Buhl EH (1998). Salient features of synaptic organisation in the cerebral cortex. Brain Res. Rev.

[84] Somogyi P, Klausberger T (2005). Defined types of cortical interneurone structure space and spike timing in the hippocampus. J. Physiol.

[85] Somogyi P, Nunzi MG, Gorio A, Smith AD (1983). A new type of specific interneuron in the monkey hippocampus forming synapses exclusively with the axon initial segments of pyramidal cells. Brain Res.

[86] Stumm RK, Zhou C, Schulz S, Hollt V (2004). Neuronal types expressing mu- and delta-opioid receptor mRNA in the rat hippocampal formation. J. Comp. Neurol.

[87] Svoboda KR, Adams CE, Lupica CR (1999). Opioid receptor subtype expression defines morphologically distinct classes of hippocampal interneurons. J. Neurosci.

[88] Thomson AM, Bannister AP, Hughes DI, Pawelzik H (2000). Differential sensitivity to Zolpidem of IPSPs activated by morphologically identified CA1 interneurons in slices of rat hippocampus. Eur. J. Neurosci.

[89] Tsou K, Mackie K, Sañudo-Peña MC, Walker JM (1999). Cannabinoid CB1 receptors are localized primarily on cholecystokinin-containing GABAergic interneurons in the rat hippocampal formation. Neuroscience.

[90] Twitchell W, Brown S, Mackie K (1997). Cannabinoids inhibit N- and P/Q-type calcium channels in cultured rat hippocampal neurons. J. Neurophysiol.

[91] Varma N, Carlson GC, Ledent C, Alger BE (2001). Metabotropic glutamate receptors drive the endocannabinoid system in hippocampus. J. Neurosci.

[92] Vásquez C, Navarro-Polanco RA, Huerta M, Trujillo X, Andrade F, Trujillo-Hernández B, Hernández L (2003). Effects of cannabinoids on endogenous K+ and Ca^2+^ currents in HEK293 cells. Can. J. Physiol. Pharmacol.

[93] Vida I, Halasy K, Szinyei C, Somogyi P, Buhl EH (1998). Unitary IPSPs evoked by interneurons at the stratum radiatum-stratum lacunosum-moleculare border in the CA1 area of the rat hippocampus *in vitro*. J. Physiol.

[94] Wang Y, Toledo-Rodriguez M, Gupta A, Wu C, Silberberg G, Luo J, Markram H (2004). Antomical, physiological and molecular properties of Martinotti cells in the somatosensory cortex of the juvenile rat. J. Physiol.

[95] Wank SA (1995). Cholecystokinin receptors. Am. J. Physiol.

[96] Westbrook GL, Lothman EW (1983). Cellular and synaptic basis of kainic acid-induced hippocampal epileptiform activity. Brain Res.

[97] Wilson RI, Nicoll RA (2002). Endocannabinoid signalling in the brain. Science.

[98] Wilson RI, Kunos G, Nicoll RA (2001). Presynaptic specificity of endocannabinoid signalling in the hippocampus. Neuron.

[99] Zieglgansberger W, French ED, Siggins GR, Bloom FE (1979). Opioid peptides may excite hippocampal pyramidal neurons by inhibiting adjacent inhibitory interneurons. Science.

[100] Zilberter Y (2000). Dendritic release of glutamate suppresses synaptic inhibition of pyramidal neurons in rat neocortex. J. Physiol.

